# MALDI-MS Imaging of Urushiols in Poison Ivy Stem

**DOI:** 10.3390/molecules22050711

**Published:** 2017-04-29

**Authors:** Mina Aziz, Drew Sturtevant, Jordan Winston, Eva Collakova, John G. Jelesko, Kent D. Chapman

**Affiliations:** 1 Department of Biological Sciences, BioDiscovery Institute, University of North Texas, Denton, TX 76203, USA; mina.aziz@unt.edu (M.A.); drewsturtevant@my.unt.edu (D.S.); 2 Department of Plant Pathology, Physiology, and Weed Science, Virginia Tech University, Blacksburg, VA 24061, USA; jordanw3@vt.edu (J.W.); collakov@vt.edu (E.C.)

**Keywords:** MALDI-MSI, *Toxicodendron radicans*, poison ivy, urushiols, in situ localization

## Abstract

Urushiols are the allergenic components of *Toxicodendron radicans* (poison ivy) as well as other Toxicodendron species. They are alk-(en)-yl catechol derivatives with a 15- or 17-carbon side chain having different degrees of unsaturation. Although several methods have been developed for analysis of urushiols in plant tissues, the in situ localization of the different urushiol congeners has not been reported. Here, we report on the first analysis of urushiols in poison ivy stems by matrix-assisted laser desorption/ionization-mass spectrometry imaging (MALDI-MSI). Our results show that the urushiol congeners with 15-carbon side chains are mainly localized to the resin ducts, while those with 17-carbon side chains are widely distributed in cortex and vascular tissues. The presence of these urushiols in stem extracts of poison ivy seedlings was confirmed by GC-MS. These novel findings provide new insights into the spatial tissue distribution of urushiols that might be biosynthetically or functionally relevant.

## 1. Introduction

Plants in the family Anacardiaceae, especially those of the genus Toxicodendron such as *Toxicodendron radicans* (poison ivy) and *Toxicodendron diversilobum* (poison oak) are well known for causing contact dermatitis in sensitive individuals [1]. The toxicodendron allergens that cause irritation, inflammation, and blistering upon skin contact are generically known as urushiols. Urushiols are lipophilic catechol derivatives with a 15- or 17-carbon unbranched alk-(en)-yl side chain that are either fully saturated or have 1–3 double bonds [2,3,4,5]. Structure-activity studies have revealed that both the catechol ring and the side chain are required for urushiols’ allergenicity; for example, the dimethyl ether derivatives are not allergenic, while the urushiol congeners with higher degree of unsaturation in the side chains have higher allergenic potential [6,7,8].

The isolation of urushiols from natural sources is technically difficult due to their high susceptibility to air oxidation and polymerization as well as their irreversible binding to the common chromatographic adsorbents [9,10]. Several methods have been developed for isolation of urushiols based on their prior derivatization [4,11,12,13], however the first isolation and spectral characterization of the pure underivatized individual urushiol congeners from both poison ivy and poison oak was reported in 1982 [10]. The first GC-MS analysis of urushiols was reported by Gross et al. (1975; [12]). Draper et al. (2002) employed atmospheric pressure ionization (API) LC-MS-MS for urushiols determination [14]. More recently, urushiols were directly detected and identified in poison ivy leaves, without any sample preparation steps, using leaf spray mass spectrometry [15]. However, the spatial tissue localization of the different urushiol congeners has not been reported, and could reveal important insights into the compartmentalization of the synthesis of urushiols. 

Matrix-assisted laser desorption/ionization-mass spectrometry imaging (MALDI-MSI) is a powerful analytical approach that has been recently employed for direct visualization of the spatial distribution of different primary and specialized metabolites in plant cells and tissues (reviewed in [16]). Here, we report on the first MALDI-MSI analysis of urushiols in poison ivy stem. Our results indicate that the in situ localization of the urushiol congeners with 15-carbon side chains is distinctly different from those with 17-carbon side chains in both the hypocotyl and first internode stem tissues. GC-MS confirmed the presence of these urushiols in stem extracts of poison ivy seedlings. These novel findings shed light on a differential tissue distribution of urushiols that might be biosynthetically or functionally relevant. 

## 2. Results

**Figure 1:**
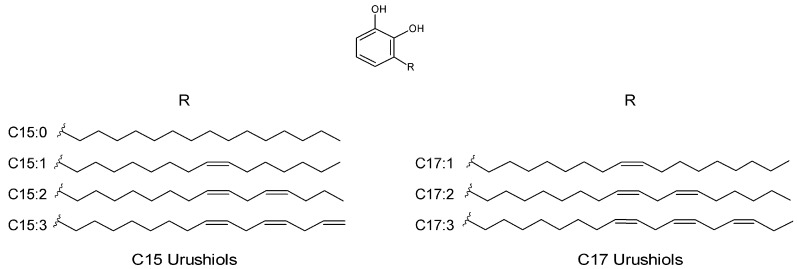
Structures of poison ivy urushiol congeners.

Urushiols are alk-(en)-yl catechol derivatives with a 15- or 17-carbon side chain that is either fully saturated or has 1–3 double bonds. The empirical formulas, monoisotopic masses, as well as the calculated masses of the corresponding sodium adducts of the different urushiol congeners in poison ivy are summarized in Table 1, with the representative structures depicted in Figure 1.

**Figure 2:**
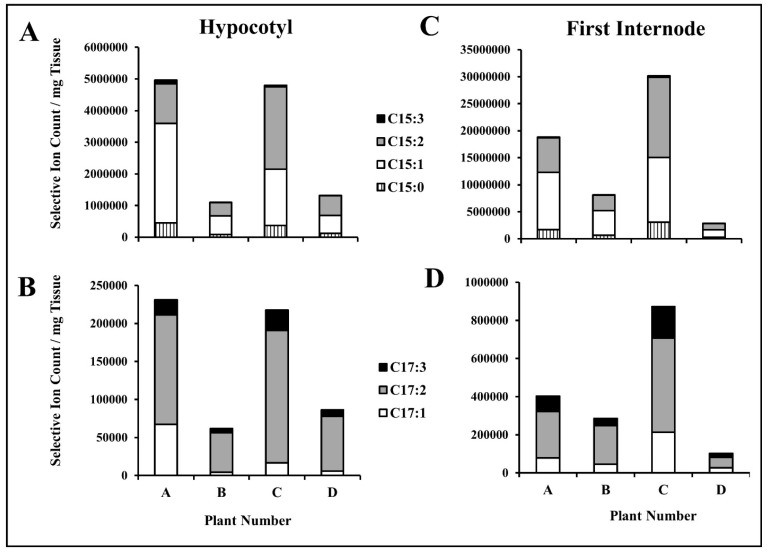
GC-MS quantification of urushiols in poison ivy stem tissues extracts. Urushiols were extracted from both hypocotyls (**A**,**B**) and the first internodes (**C**,**D**) collected from four individual five-week-old plants using C15:0 alkylresorcinol as an internal standard and the different C15 and C17 urushiol congeners were quantified using GC-MS and specific fragments as described in Material and Methods. There is a considerable variability in the total urushiol content among the four individual plants, however the relative abundance of the different congeners is quite similar, where C15:1 and C15:2 are the most abundant among all C15 species, while C17:2 is the most abundant C17 congener.

GC-MS analysis of poison ivy extracts of four different hypocotyls and stem (first internodes) samples showed that the total urushiol content was higher in the first internodes than the hypocotyls (Figure 2). Although there was a considerable variability in the total urushiol content among individual plants, the relative proportion of the different urushiol congeners was more or less similar in all tissues examined; pentadecenylcatechol (C15:1) and pentadecadienylcatechol (C15:2) are the most abundant C15 species, while heptadecadienylcatechol (C17:2) is the most abundant C17 congener (Figure 2). Relative quantification was based on a C15 alkylresorcinol added at the time of extraction as an internal standard.

**Figure 3:**
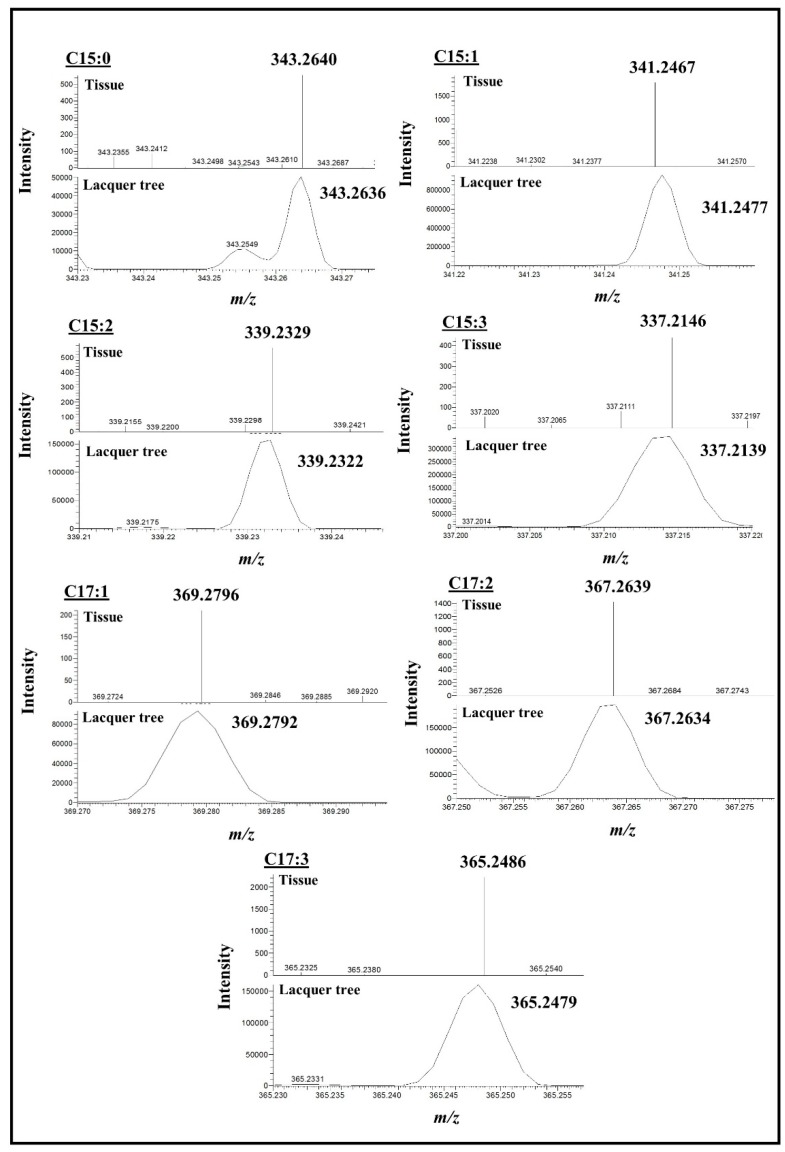
Selected regions of MALDI-MS spectra containing urushiol sodium adducts. Identification of urushiol sodium adducts was made in MALDI-MS imaging datasets acquired for both poison ivy stem sections and the Japanese lacquer tree extract. Identified ions had very similar masses in both the sections and the extract with the mass difference between the theoretical and observed masses is less than 10 ppm for all selected ions. The MALDI-MSI data were collected in centroid and profile modes for the tissue sections and the Japanese lacquer tree extract, respectively. A broader mass range is depicted in supplementary Figures S1 and S2 for one of the Japanese lacquer tree extracts and one of the stem tissue sections, respectively, where the C15:1 urushiol congener was identified.

To assess the location of urushiols in poison ivy seedling, tissue sections from three-week-old plants were examined by MALDI-MSI. For an urushiol standard, and to evaluate the behavior of the molecules in MALDI-MS, extracts obtained from the Japanese lacquer tree (*Toxicodendron vernicifluum*) were also analyzed by MALDI-MS. The expected *m*/*z* ions corresponding to Na^+^ adducts of the different urushiols were identified in both the Japanese lacquer tree extracts and in sections of poison ivy seedling tissues (Figure 3). Identified *m*/*z* values were very similar in both the tissue sections and in standard urushiol extracts with differences between the theoretical and observed *m*/*z* are less than 10 ppm for all identified ions (Figure 3). 

**Figure 4:**
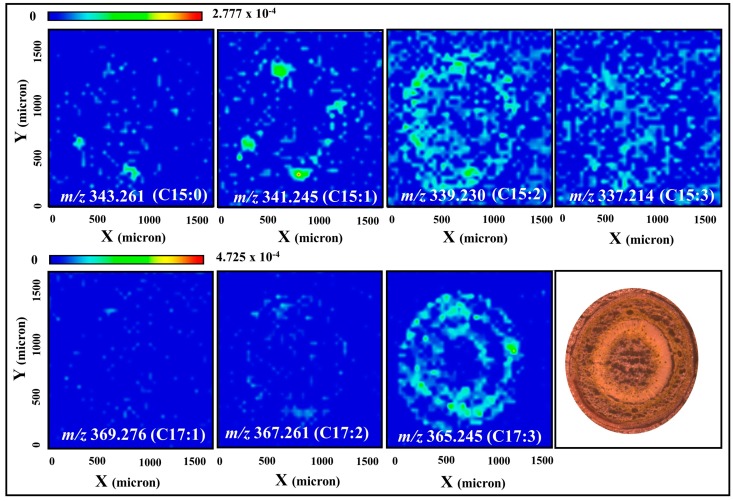
Spatial localization of urushiols in poison ivy hypocotyl sections. Sodium adduct of the C15:1 congener is localized to the resin ducts while that of the C17:3 is distributed predominantly in the cortex and vascular tissues of the hypocotyl. Distribution of the other congeners is not well-defined. Intensity of the selected ions was normalized to the total ion count before the images were generated.

**Figure 5:**
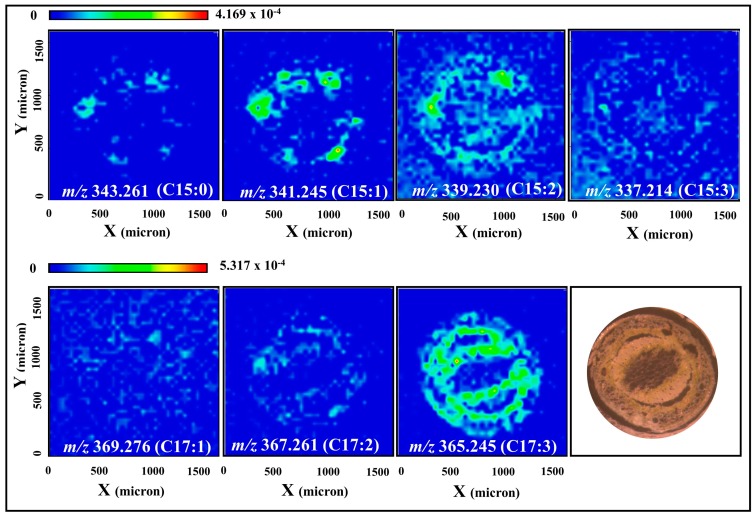
Spatial localization of urushiols in poison ivy stem (first internode) sections. Sodium adducts of the C15:0, C15:1, and C15:2 congeners are localized to the resin ducts. In contrast, C17:2 and C17:3 congener sodium adducts are localized to the cortex and vascular tissues of the stem. Distribution of the other congeners is not well-defined. Intensity of the selected ions was normalized to the total ion count prior to image generation.

**Figure 6:**
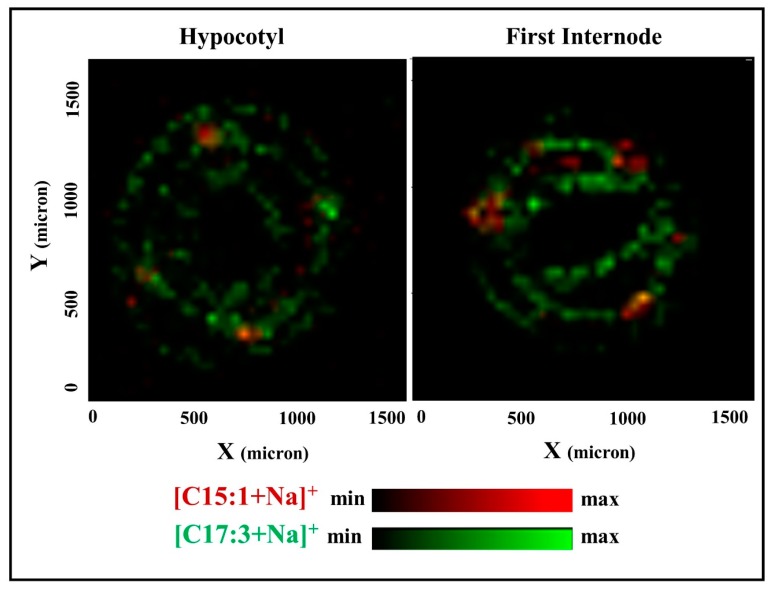
The C15 and C17 urushiol congeners have distinctly different localization within poison ivy hypocotyls and the first internodes. Combined images showing that the sodium adduct of the C15:1 congener (red) is mainly localized to the resin ducts, while that of the C17:3 congener (green) is more widely distributed in the cortex and vascular tissues in both the hypocotyl and the first internode regions of the stem. These images were generated by merging the individual images of the selected ions (*m*/*z*) presented in Figure 4 and Figure 5.

MALDI-MSI analysis of poison ivy sections revealed distinctly different tissue localization for the C15 and C17 urushiol species. For example, in hypocotyls, the sodium adduct of the C15:1 urushiol congener was exclusively localized to the resin ducts, while that of the C17:3 congener was widely distributed in the cortex and vascular tissues (Figure 4). A similar pattern was observed in the first internode stem tissues, where the [M + Na]^+^ ions of the C15:0, C15:1 and C15:2 urushiol congeners are localized to the resin ducts, and those of the C17:2 and C17:3 congeners were mostly distributed in the cortex and vascular tissues of the stem (Figure 5). False-color merged images demonstrate the distinctly-different tissue distribution patterns of the C15:1 and C17:3 urushiol congeners in both hypocotyls and the first internode stems (Figure 6).

## 3. Discussion

Urushiols are the allergenic components of poison ivy. They are alk-(en)-yl catechol derivatives with a 15- or 17-carbon side chain having different degrees of unsaturation. Several methods have been developed for analysis of urushiols in plant extracts, resulting in the loss of information about the spatial tissue distribution of these metabolites. In the current study, we directly visualized the tissue localization of the different urushiol congeners in poison ivy hypocotyls and first-internode stems using MALDI-MSI. Our results revealed that the urushiol congeners with 15-cabon side chains are mainly localized to the resin ducts, while those with 17-carbon side chains are more widely distributed in cortex and vascular tissues. The spatial separation of 15-carbon from 17-carbon urushiols was an unexpected finding because proposed urushiol biosynthetic pathways [17,18] have not considered the possibility that pentadecyl- and heptadecyl-urushiol might originate in different cell types. The Japanese lacquer tree (*T. vernicifluum*) and poison ivy (*T. radicans*) produce nearly identical urushiol congeners (differing only in the location of one vinyl group in the pentadecatrienyl-urushiol congeners [19]). In the Japanese lacquer tree, urushiol sap yields are proportional to the thickness of the bark, along with the number and diameter of resin ducts therein [20]. Thus, it was generally assumed that all urushiol accumulation and biosynthesis in the genus Toxicodendron is associated with resin canals/ducts. However, our findings suggest the possibility that C15- and C17-urushiols maybe synthesized in different cell types, at least in poison ivy. However, because MALDI-MSI detects spatially resolved metabolites due to steady state accumulation levels, it is also formally possible that all urushiols are synthesized in one cell type and then are differentially transported to other cell types where they accumulate. Additional studies will be required to resolve the biosynthetic routes and sources for urushiols in poison ivy stem tissues.

There are enormous gaps in knowledge about urushiol synthesis. The long alk-(en)-yl chain suggests that urushiols are derived from fatty acid metabolism. First Giessman (1967; [17]) and then later DeWick (1997; [18]) proposed that urushiol synthesis begins with a polyketide synthase activity to create a fatty acid tetraketide of which one keto group is reduced and then undergoes an aldol condensation, decarboxylation, and hydroxylation to generate an alkyl-catechol aromatic ring. To date, none of the proposed enzymatic activities have been characterized. This study and others [12,21] identified much higher levels of C15- urushiols than the C17-urushiols in poison ivy. Likewise, the relative composition of unsaturated C17-urushiol congeners differed from the relative abundance of unsaturated C15-urushiol congeners. It is not known if the unsaturation is derived from the fatty acid-CoA starter used by a polyketide synthase or a desaturase enzyme acting after the formation of the catechol ring. In either scenario, posited cell-type-specific expression of different members of polyketide synthase and/or desaturase multigene families would be consistent with our observed spatial separation of C15- and C17-urushiol congeners with different degrees of unsaturation. 

Previous reports of poison ivy urushiol-relative congener composition are often conflicting. This is likely due to uncontrolled accession and environmental parameters that arise from collecting wild poison ivy plant material from different locations. To the best of our knowledge, this study is the first report evaluating urushiol levels using poison ivy seedlings (derived from drupes collected at a particular location) and cultivated under axenic, environmentally controlled conditions [22]. Even with this unprecedented level of environmental control, the four poison ivy seedlings evaluated by GC-MS showed substantial variation in total urushiol levels, and—to a much lesser degree—differences in relative urushiol congener composition. Thus, considerable urushiol variability was observed despite controlling for all biotic and abiotic environmental effects. Thus, the most likely source of variation was due to individual seedling-level genotypic effects. In this regard, it is noteworthy that poison ivy is an obligate out-crossing dioecious species, where the potential for allelic heterozygosity is high. The drupes used in the GC-MS analyses were collected from one location but from an unspecified number of different female lianas, pollinated by unknown male plants. It is also worth considering that the degree to which urushiol levels and/or composition is subjected to natural selection is currently an enigmatic topic (see discussion below). 

MALDI-MSI has been applied to directly visualize plant tissues, where the recorded mass spectra at every spot can be used to generate ‘heat maps’ that correlate the observed abundances of ions with their tissue localizations. Therefore, MALDI-MSI is invaluable qualitative analytical tool to determine the spatial distribution of different primary and secondary metabolites in plant cells and tissues [16]. However, metabolite quantification has been challenging to achieve in MALDI-MSI, mainly due to the ”pixel-to-pixel variability” that can result from different sources including morphological characteristics, ionization efficiency, and detection efficiency [23,24]. It is best to compare relative abundances in MALDI-MSI with quantitative estimates from extracts, like we have done here with GC-MS. For GC-MS analysis, a C15 alkylresorcinol internal standard was added at the time of extraction and used (with an appropriate standard curve) to quantify the urushiols in the stem extracts. Comparing the relative abundance of the different urushiol congeners using GC-MS and MALDI-MSI shows that the relative abundance of the C15 species is consistent between both analyses. In contrast, for the C17 species, C17:2 is the predominant urushiol congener in GC-MS, while C17:3 is the most abundant in MALDI-MSI. This observed inconsistency may have resulted from differential ionization of the C17:2 and C17:3 urushiol congeners in MALDI-MSI. Another possibility is the different age of poison ivy seedlings used for GC-MS (five-week-old) and MALDI-MSI (three-week-old), where the relative composition of the C17 urushiol congeners may change with the age of the plant. In brief, conclusions should be reserved about metabolite quantification based on MALDI-MSI because of the aforementioned reasons; however, MALDI-MSI analysis of poison ivy is an irreplaceable methodology to detect the unique in situ localization of the different urushiol congeners revealed in the current study.

Another considerable gap in knowledge about poison ivy biology is urushiol chemical ecology. To date, only humans are documented to show substantive discomfort upon exposure to or ingestion of poison ivy plant material [25]. Deer, goats, mice, and other mammals readily eat poison ivy foliage without apparent discomfort [26,27,28]. A number of native birds eat ripe poison ivy drupes, again without signs of discomfort [27,29]. Nevertheless, all poison ivy organs accumulate urushiol, suggesting that urushiol is a chemical defense [30]. To this end, the spatial/cell-type-specific urushiol congener accumulation patterns may indicate chemical defense against different ecological target organisms. The accumulation of chemical defenses in resin canals is often a defense against chewing herbivores causing tissue disruption, and the release of a preformed chemical defense. A particularly germane example of this in the Anacardiaceae is the accumulation of closely related alkylresorcinols in resin canals of mango fruit peels that correlate with resistance to insect pests [31,32]. On the other hand, the accumulation of C17-urushiols in epidermis, cortex, and vasculature may be a chemical defense (phytoalexin) directed towards microbial pathogens. Additional research will be required to elucidate the biological significance of urushiols and the environmental factors influencing their accumulation.

## 4. Materials and Methods 

### 4.1. Plant Growth Conditions 

*Toxicodendron radicans* drupes were collected from the Virginia Tech Golf Course (VAMontCo-1) in Montgomery County Virginia and VARoaCo-1 [22]. The drupes were subjected to a combination of mechanical, chemical, and sterile plant culture methods resulting in individual axenic poison ivy seedlings growing on half-strength MS media in Magenta boxes (Sigma, St. Louis, MO, USA) [22]. Seedlings were grown at constant 28 °C under a 16-h light cycle in a Percival CU-36l4 environmental chamber (Percival Scientific, Perry, IA, USA). Seedlings used for MALDI-MSI were grown for three weeks and those used for GC-MS were grown for five weeks to ensure sufficient dry biomass for accurate measurement of internode material.

### 4.2. GC-MS Urushiol Extraction and Quantification

A biphasic metabolite extraction method was adapted from a polar and non-polar metabolite profiling protocol [33] to efficiently extract urushiols and analyze their steady-state levels. This protocol is based on an optimized and widely-used metabolomics extraction procedure to separate polar (sugars, amino acids, carboxylic acids, etc.) and non-polar metabolites (fatty acids, chlorophylls, carotenoids, sterols, tocopherols, etc.) for GC-MS analysis in a variety of plant tissues and organs [34]. Consisting of a polar catechol head-group and a non-polar hydrocarbon tail, urushiols are structurally similar to tocopherols consisting of a polar chromanol head-group and a non-polar prenyl tail. Therefore, this biphasic extraction method used for analyzing these non-polar antioxidants is suitable for urushiol analysis. Lyophilized and powdered plant material (2–5 mg) was weighed on a Mettler Toledo (Columbus, OH, USA) XS205 Dual Range analytical balance that accurately measures within this range. The lyophilized poison ivy VAMontCo-1 plant material was extracted using a biphasic metabolite extraction and derivatization procedure as described in Collakova et al. [33], except that 1 mg/mL of butylated hydroxytoluene in chloroform was also used to prevent urushiol oxidation during extractions, as in the case of preventing tocopherol oxidation [35]. Two ng of C15:0 pentadecyl-resorcinol (SIGMA, Saint Louis, MO, USA) was used as an internal standard because it differs from pentadec(en)yl-catechols only in the relative positions of the two hydroxyl groups (meta vs. ortho, respectively) and elutes at a unique retention time from all pentadec(en)yl- and heptadec(en)yl-urushiols. This biphasic metabolite extraction procedure [33] resulted in complete extraction of non-polar compounds into the chloroform phase, as evidenced by the white insoluble material present at the organic-aqueous interphase, indicating a complete chlorophyll extraction and partitioning to the organic phase. A measure of 100 microliters of the urushiol-containing chloroform phase was evaporated at 50 °C under a stream of nitrogen gas and urushiols were derivatized in the presence of 2,2,2-trifluoro-*N*-methyl-*N*-(trimethylsilyl)-acetamide containing 1% trimethylchlorosilane diluted 1:1 (*v*:*v*) with pyridine (Thermo Fisher Scientific, Waltham, MA, USA). Trimethylsilylated (TMS) urushiol derivatives (1 µL) were injected in a pulsed splitless mode onto an Agilent 7890 A series GC equipped with a DB-5MS-DG column (30 m length × 0.25 mm × 0.25 μm with a 10-m pre-column, Agilent Technologies, Santa Clara, CA, USA) and analyzed on an Agilent 5975 C series single quadrupole MS. The GC separation was achieved by holding the temperature at 170 °C for 1 min and followed by temperature ramping at a rate of 10 °C/min from 170 to 320 °C. The resulting 20-min GC separation program was concluded by a 4-min hold at 320 °C. The MS parameters were as follows: the MS source and quad were kept at 250 and 150 °C, respectively. The *m*/*z* ratio range was from 100 to 510 and sampling was done at a rate of 11.57 scans/s. Pentadec(en)yl urushiols with differing degrees of unsaturation share two major common fragments in their MS spectra (*m*/*z* 179.0 and 267.1, indicating one and two TMS groups at the *o*-hydroxyls, respectively), but the individual congeners are distinguished based on their molecular ions as well as the ions corresponding to the loss of a methyl group (M-15) from the molecular ions. The molecular ions of the individual TMS-derivatized urushiols were previously determined [12,36]. These molecular ions were used to obtain peak areas for the individual urushiols. Peak area analysis was performed by using the Enhanced Mass Selective Detector ChemStation software (Agilent Technologies). Peak areas for each urushiol were normalized to dry weight and extraction efficiency to obtain relative steady-state urushiol congener levels in each seedling tested. An alkylresorcinol standard curve (0.0, 0.1, 0.5, 1.0, 5.0, and 10.0 ng alkylresorcinol) was processed along with the poison ivy stem section samples to support relative quantification of urushiols in extracts with the assumption that there were similar ionization efficiencies between this structurally similar standard and the respective analytes of interest.

### 4.3. Cryo-Sectioning for MALDI-MS Imaging 

Axenic poison ivy hypocotyl and first internode sections were dissected from axenic poison ivy (VARoaCo-1) seedlings with scissors and then embedded in molten (40 °C) 8% porcine gelatin, and then solidified at room temperature. The embedded samples were incubated at −20 °C for five minutes and transferred to −80 °C where they were incubated overnight. The gelatin embedded samples were transferred back to −20 °C for a duration of four days. The frozen samples were transferred to a Microm HM550 Cryostat (Thermo Fisher Scientific, Kalamazoo, MI, USA) set at −17 °C, where they were cut into 25 µm cross sections, mounted on glass slides, and lyophilized overnight. The lyophilized samples were immediately placed in 50 mL screw-capped Falcon tubes, placed into hermetically-sealed plastic bags containing desiccant packs, and then sent by overnight courier to the Chapman lab, where they were analyzed by MALDI-MS.

### 4.4. MALDI-MS Imaging Analysis

The MALDI-MSI method was similar to that described in Woodfield et al. (2017; [37]). Lyophilized poison ivy stem sections were uniformly coated by 2,5-dihydroxybenzoic acid (DHB) matrix via sublimation. Tissue sections were analyzed on a MALDI-LTQ Orbitrap-XL (Thermo Fisher Scientific, San Jose, CA, USA) equipped with a nitrogen laser, using 12 µJ/pulse laser energy and 40 µm spatial resolution (raster size). Data acquisition was performed in positive ion mode with an *m*/*z* range of 275–800, using the high-resolution (60,000) Orbitrap mass analyzer. Attenuated gain control (AGC) feature was enabled, which means that the instrument automatically adjusted the number of laser shots to produce the same number of ions for each scan. Three sections from three different plants were analyzed for both the hypocotyl and the first-internode stem tissues to confirm urushiol localizations. Urushiols were detected as [M + Na]^+^ ions with a mass tolerance less than 10 ppm for all selected ions. For an urushiol standard, and to evaluate the behavior of the molecules in MALDI-MS, extracts obtained from the Japanese lacquer tree (*T. vernicifluum*) were also spotted on a glass slide, coated by DHB matrix via sublimation and analyzed by MALDI-MS using the same conditions as with the tissue sections. MALDI-MS images were generated using ImageQuest software (Thermo Fisher Scientific, San Jose, CA, USA); intensity of the selected ions were normalized to the total ion count before generating the images. 

## Figures and Tables

**Table 1:** Summary of the different urushiol congeners in poison ivy.

Compound	Abbreviation	Empirical Formula	Monoisotopic Mass	[M + Na]^+^
Pentadecylcatechol	C15:0	C_21_H_36_O_2_	320.2715	343.2613
Pentadecenylcatechol	C15:1	C_21_H_34_O_2_	318.2558	341.2456
Pentadecadienylcatechol	C15:2	C_21_H_32_O_2_	316.2402	339.2300
Pentadecatrienylcatechol	C15:3	C_21_H_30_O_2_	314.2245	337.2143
Heptadecenylcatechol	C17:1	C_23_H_38_O_2_	346.2871	369.2769
Heptadecadienylcatechol	C17:2	C_23_H_36_O_2_	344.2715	367.2613
Heptadecatrienylcatechol	C17:3	C_23_H_34_O_2_	342.2558	365.2456

## Supplementary Materials

Supplementary materials are available online. Figure S1: MALDI-MSI spectrum of the Japanese lacquer tree extract within an *m*/*z* range of 335–350, Figure S2: MALDI-MSI spectrum of poison ivy stem tissue section within an *m*/*z* range of 335–350.
