# Supplementary material for: MALDI-MS Imaging of Urushiols in Poison Ivy Stem
Source: Molecules. 2017 Apr 29;22(5):711. doi: 10.3390/molecules22050711 (PMC6154699; doi:10.3390/molecules22050711)
Supplement: Supplementary file 1 [file molecules-22-00711-s001.pdf]

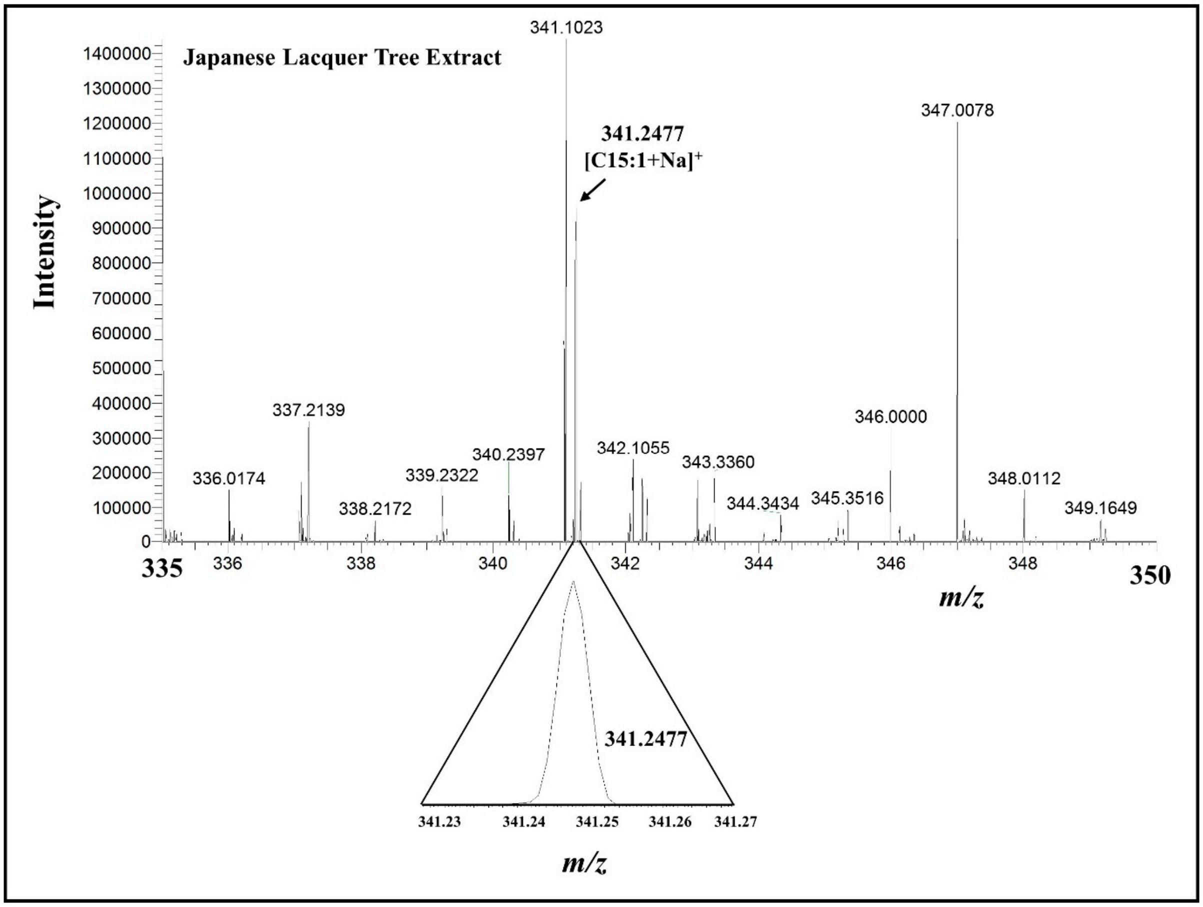

Figure S1. MALDI-MSI spectrum of the Japanese lacquer tree extract within an *m/z* range of 335-350. An example of MALDI-MSI spectrum of the Japanese lacquer tree extract within an *m/z* range of 335-350 with a region zoomed in to show *m/z* of 341.2477 corresponding to the sodium adduct of the C15:1 urushiol congener. The MALDI-MSI data were collected in profile mode for the Japanese lacquer tree extract.

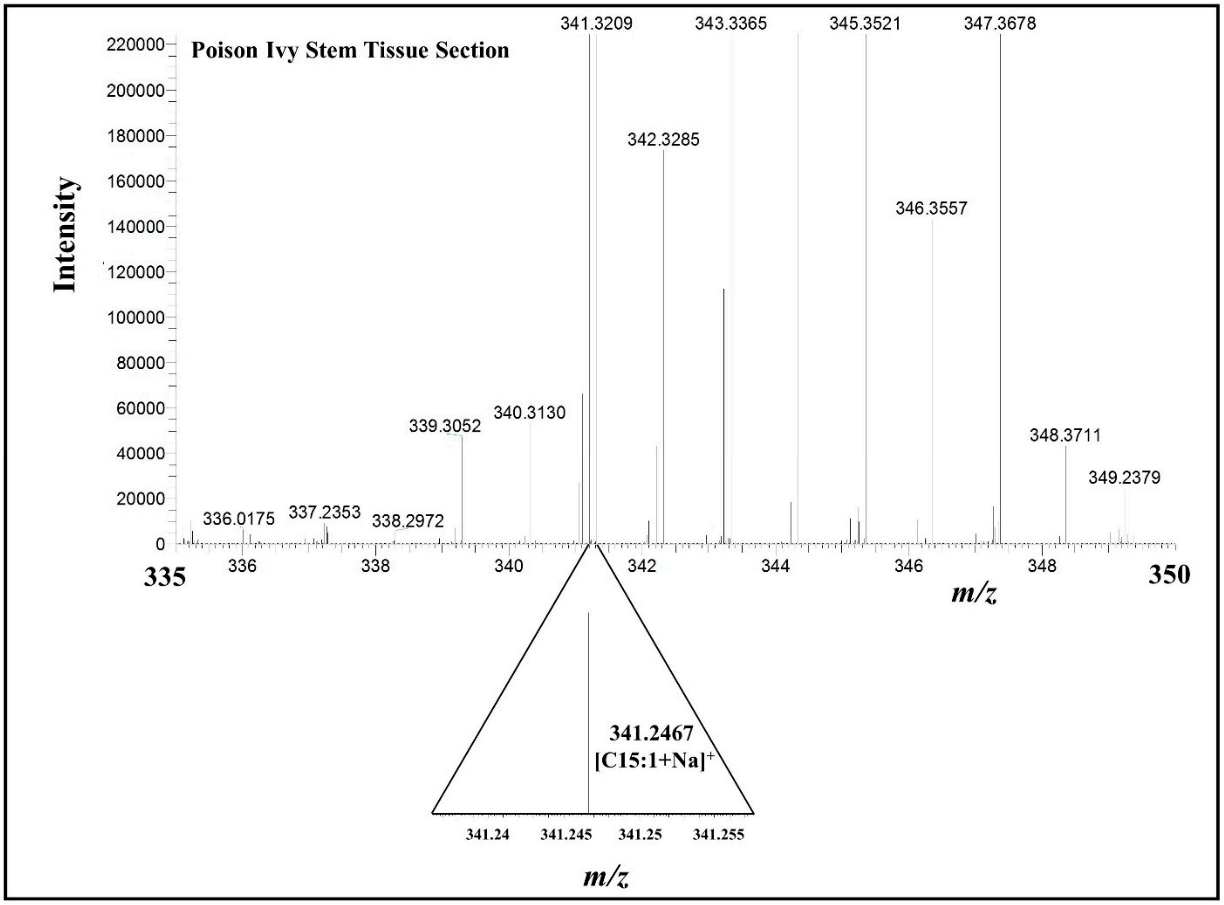

Figure S2. MALDI-MSI spectrum of poison ivy stem tissue section within an *m*/*z* range of 335-350. An example of MALDI-MSI spectrum of poison ivy stem tissue section within an *m*/*z* range of 335-350 with a region zoomed in to show *m*/*z* of 341.2467 corresponding to the sodium adduct of the C15:1 urushiol congener. The MALDI-MSI data were collected in centroid mode for the poison ivy stem tissue section.
